# Association between Hypertension and Chronic Arsenic Exposure in Drinking Water: A Cross-Sectional Study in Bangladesh 

**DOI:** 10.3390/ijerph9124522

**Published:** 2012-12-07

**Authors:** Mohammad Rafiqul Islam, Ismail Khan, John Attia, Sheikh Mohammad Nazmul Hassan, Mark McEvoy, Catherine D’Este, Syed Azim, Ayesha Akhter, Shahnaz Akter, Sheikh Mohammad Shahidullah, Abul Hasnat Milton

**Affiliations:** 1 Centre for Clinical Epidemiology & Biostatistics (CCEB), The University of Newcastle, Lot 1 Kookaburra Circuit, New Lambton Heights, NSW 2305, Australia; E-Mails: mdrafiqul.islam@newcastle.edu.au (M.R.I.); John.Attia@newcastle.edu.au (J.A.); Mark.McEvoy@newcastle.edu.au (M.M.); Catherine.dEste@newcastle.edu.au (C.D.); 2 Department of Pharmacology, Dhaka Medical College, Dhaka 1000, Bangladesh; E-Mail: ismailbangladesh@yahoo.com; 3 Department of Public Health, Atish Dipankar University of Science & Technology, Dhaka 1205, Bangladesh; E-Mail: nazmulhena@yahoo.com; 4 School of Public Health and Community Medicine, University of New South Wales, Sydney, NSW 2052, Australia; E-Mail: s.azim@unsw.edu.au; 5 Department of Obstetrics and Gynaecology, Tairunnessa Memorial Medical College, Targas, Kunia, Gazipur, Dhaka, Gazipur 1701, Bangladesh; E-Mail: akhterayesha@yahoo.com.au; 6 Department of Paediatrics, Institute of Child and Mother Health (ICMH), Matuail, Demra, Dhaka 1362, Bangladesh; E-Mail: nupur.dmc56@gmail.com; 7 NGO Forum for Drinking Water, Lalmatia, Dhaka 1207, Bangladesh; E-Mail: smshahidullah@gmail.com

**Keywords:** arsenic, drinking-water, hypertension, pulse-pressure, Bangladesh

## Abstract

Chronic arsenic exposure and its association with hypertension in adults are inconclusive and this cross-sectional study investigated the association. The study was conducted between January and July 2009 among 1,004 participants from 1,682 eligible women and men aged ≥30 years living in rural Bangladesh who had continuously consumed arsenic-contaminated drinking water for at least 6 months. Hypertension was defined as systolic blood pressure ≥140 mmHg (systolic hypertension) and diastolic blood pressure ≥90 mmHg (diastolic hypertension). Pulse pressure was calculated by deducting diastolic from systolic pressure and considered to be increased when the difference was ≥55 mmHg. The prevalence of hypertension was 6.6% (95% CI: 5.1–8.3%). After adjustment for other factors, no excess risk of hypertension was observed for arsenic exposure >50μg/L or to that of arsenic exposure as quartiles or as duration. Arsenic concentration as quartiles and >50 μg/L did show a strong relationship with increased pulse pressure (adjusted OR: 3.54, 95% CI: 1.46–8.57), as did arsenic exposure for ≥10 years (adjusted OR: 5.25, 95% CI: 1.41–19.51). Arsenic as quartiles showed a dose response relationship with increased pulse pressure. Our study suggests an association between higher drinking water arsenic or duration and pulse pressure, but not hypertension.

## 1. Introduction

Arsenic is a toxic metalloid that is found in various compounds in the soil and extensively distributed in the Earth’s crust [1,2]. Exposure to inorganic arsenic via drinking water, occupation, medication, and other sources has wide ranging effects on health conditions, based on data from many parts of the World [1,3,4,5]. These health effects range from skin lesions, stillbirth, spontaneous abortion, poor nutritional status during pregnancy, cardiovascular diseases, diabetes to cancers of many types [6,7,8,9,10,11,12,13]. On the other hand, cessation of arsenic contaminated artesian well water consumption in Taiwan decreased the mortality from peripheral vascular disease and ischemic heart disease [14,15] and arsenic exposure is likely to be causal of ischemic heart disease related mortality [15]. Ingested inorganic arsenic may directly affect the atherogenic process involving vascular endothelium, smooth muscle cells, platelets and macrophages and can cause black foot disease and ischemic heart disease; arsenic may also indirectly affect many risk factors for cardiovascular diseases [1]. 

Among the cardiovascular risk factors, hypertension is a well-known and widespread. Hypertension is generally defined as abnormally high arterial blood pressure indicated by an adult systolic blood pressure of 140 mmHg or greater or a diastolic blood pressure of 90 mm Hg or greater [16]. In addition to general hypertension, an increased risk of vascular disease is associated with many other blood pressure dimensions such as systolic (SBP), diastolic (DBP), mean arterial (defined as ⅓ × (SBP + 2 × DBP)) and pulse pressure (the difference between SBP and DBP) [17]. Globally, approximately one billion adult people are suffering from hypertension, of which 66% are from developing countries [18]. In the industrialized countries, it is one of the most important risk factors for death [19]. About 90–95% of hypertensive cases are suffering from primary or essential hypertension while the rest are from secondary causes such as reno-vascular and endocrine disorders [20,21,22]. Important known associated risk factors for primary hypertension are sedentary lifestyles, hypokalaemia, obesity, higher body mass index (BMI), sensitivity to table salt, high alcohol consumption, ageing, having a positive family history, low birth weight, insulin resistance and metabolic syndrome [23,24,25,26,27,28,29,30,31,32]. 

Chronic arsenic toxicity via drinking water consumption has been significantly associated with hypertension in many ecological studies. Chronic arsenic exposure was associated with increased systolic and diastolic blood pressure in a village population in Iran [33]. One Indian study showed higher liver arsenic concentrations among those who drank arsenic contaminated water and reported a possible association with idiopathic portal hypertension [34]. A few studies conducted in Taiwan reported dose response relationships of chronic arsenic exposure with hypertension and cerebrovascular diseases [1,35]. A Bangladeshi study estimated a doubling in the risk of being hypertensive in people exposed to one milligram of arsenic in each litre, compared to less than 0.5 milligram per litre of drinking water [36]. Another Bangladeshi study reported no association of arsenic to overall and diastolic hypertension but observed an association with increased pulse pressure and systolic hypertension and recommended further studies to investigate the relationship of low-levels of drinking water arsenic exposure with atherosclerosis and cardiovascular diseases [17]. However, the dose response relationship and toxicological mechanism of arsenic on hypertension are still unclear [37]. Therefore, we aimed to evaluate hypertension in rural Bangladeshi adults on or over 30 years of age exposed to various levels of drinking water arsenic at least for a period of six months.

## 2. Methods

### 2.1. Overview

Bangladesh is divided into 64 districts, with each district divided into sub-districts named upazilas, and each upazila further divided into a number of unions. Each union consists of a number of villages, each with a number of tube wells from which the inhabitants draw their water for daily usage including drinking and cooking. Ethics approval was obtained from the Bangladesh Medical Research Council (BMRC) and the Human Research Ethics Committee (HREC), The University of Newcastle, Australia.

### 2.2. Design, Study Area and Population

This cross sectional study was conducted between January and July 2009 in Bangladesh. We reviewed the Bangladesh Arsenic Mitigation Water Supply Project’s (BAMWSP) national survey report and arsenic contaminated areas were selected. Four unions of Laksam upazila and one union of Monohargonj upazila of Comilla district were chosen as high; two unions of Kaliganj upazila of Jhenidah district were chosen as low arsenic contaminated areas. A union having ≥40% of the tube wells contaminated with arsenic >50 μg/L, of which 25% had arsenic more than 250 μg/L were considered as a high arsenic contaminated area. A union with no tube well containing arsenic at or more than 50 μg/L was considered as a low arsenic contaminated area. These areas were chosen purposively to ensure the study population’s exposure to a wide range of arsenic that enabled us to study the effect of various levels of arsenic on hypertension. Comilla and Jhenidah districts are 100 km south-east and 180 km south-west from Dhaka city, the capital of Bangladesh, respectively. One hundred households were randomly selected from each of the seven unions. All individuals present during the household visit who fulfilled eligibility criteria were recruited in the study after obtaining their informed verbal consent. Eligibility criteria were: aged ≥30 years, history of drinking water from any tube well for at least 6 months continuously, and resident in the study area for at least the preceding 6 months. 

### 2.3. Data Collection Procedure

Trained field workers conducted a door to door survey to identify potential participants from the randomly selected households. Since the literacy rate was low, the study information sheet was read directly to the potential participants, and the objectives and expectations of the study were comprehensively explained. Field workers then obtained informed verbal consent. 

Interviewers collected information on socio-demographics, and drinking water use history using a structured questionnaire. The questionnaire was developed by the researchers and pretested in a neighbouring village similar to the study area. All participants underwent a full body examination by a physician under sunlight to investigate the presence of arsenical skin lesions, *i.e.*, melanosis, leucomelanosis and keratosis. Standing height and weight of each participant were measured with the subjects wearing light clothes and no shoes. Weight was measured in kilograms using bathroom scales (Precision 500 gm) that were calibrated daily and zeroed before each measurement. Height was measured in centimetres using a locally made wooden height board with a metal measurement tape attached. 

### 2.4. Exposure Measurement

A single well water measurement was used to characterize chronic arsenic exposure for each participant. In the case of participants who changed their drinking source following BAMWSP survey, we collected water samples from the previous tubewell and duration of use. Cumulative arsenic exposure was calculated by multiplying the arsenic content of the tube well with duration of use. Drinking water samples were transferred and analysed following standard procedure [38]. Water arsenic analyses were performed at a Water Quality Testing Laboratory in Dhaka using flow injection-hydride generation atomic absorption spectrometry (FIHG-AAS) [38]. Minimum detection level of arsenic by this method is 3 μg/L. 

### 2.5. Outcome Definition and Measurement

In this study we defined overall hypertension as a systolic blood pressure ≥140 mmHg (or systolic hypertension) combined with a diastolic blood pressure ≥90 mmHg (or diastolic hypertension), as used previously in other studies [36,39]. We measured blood pressure from a participant in sitting position and in a calm and quiet environment after 15 min. This procedure followed according to the recommendation by the World Health Organization [40]. Each participant’s blood pressure was measured for 3 times on the day of visit, and for this study we considered the lowest measured value. For those with known hypertension and on antihypertensive medication (n = 12), we used the median pressure of the newly diagnosed hypertensive cases in this study. We also calculated pulse pressure by deducting diastolic from systolic pressure and defined increased pulse pressure if the difference was ≥55 mmHg [17].

### 2.6. Other Variables

Information on socio-demographic characteristics such as age, sex, income, education and occupation, marital status, drinking water history, smoking history, height, weight, arsenical skin lesions, family history of hypertension and diabetes were collected. Body Mass Index (BMI) was also calculated using the standard method (weight in kg/(height in meter)^2^) [41].

### 2.7. Statistical Analysis

Data were analysed using STATA version 10 supplied by STATA Corporation, TX, USA. Frequency tables and summary statistics were obtained to check missing data, out of range values and to assess distributions of continuous variables; logic checks were also undertaken. Water arsenic level was used to characterise chronic arsenic exposure. Arsenic concentration was categorised by the maximum acceptable limit in drinking water in Bangladesh, *i.e.*, ≤50 μg/L and >50 μg/L and also as quartiles; for each arsenic exposure categories, duration of exposure was categorised as ≤10 years and >10 years.

Multiple logistic regressions were done to determine the association between chronic arsenic exposure and hypertension, adjusting for potential confounders. Potential confounders such as participant’s age, sex, education, occupation, marital status, religion, total number of household members, income, smoking history, self-reported diabetes and body mass index (<18.2 as malnourished and ≥18.2 as normal) were included in the initial model. Only those variables found to be significant at *P* < 0.25 level of significance in the initial model were included in the final multiple regression model. We also used arsenic quartiles and modeled multiple logistic regressions to determine the association of higher levels of arsenic to that of hypertension. Cuzick’s nonparametric test for trend was used to test the trend across ordered groups.

## 3. Results

This study was conducted in 1,004 consenting women and men from 1,682 eligible participants yielding a participation rate of 60%. Approximately two third of the participants were female housewives and about 69% of the participants did not have any formal education. The participants’ mean age and BMI were 44.9 years (±13.2) and 20.3 (±4.5), respectively and nearly 99% of them were ever married. About 33% of the participants had a lean BMI (<18.22) and a similar proportion were employed. Average monthly household income was 59 US$ (±56, 1US$ = 80 Taka in Bangladesh currency). Of the participants, 14.2% were current smoker with a mean duration of smoking of 22.6 years (±12.9). Participants were mostly Muslim (77%) and 39% of the participants had different forms of arsenical skin lesions. Age and sex distributions of participants across the study areas were similar, although other socio-demographic characteristics such as education, monthly household income, BMI, arsenic concentration in drinking water, duration of arsenic exposure and history of hypertension differed substantially. About 50% of the Hindu participants and 29.5% of the Muslims were obese (BMI > 21). Arsenic concentration in the drinking water ranged from 10 to 1,401 µg/L and 46.4% of the survey household’s drinking water contained arsenic >50 ug/L. 

**Table 1:** Mean values of systolic blood pressure, diastolic blood pressure and pulse pressure.

	Overall (n = 1004)			SBP *		DBP *		Pulse blood pressure *	
	No	%	Mean As Conc* (µg/liter, SD)	Mean (mmHg, SD)	Systolic hypertension (SBP, ≥140 mmHg), %Ɨ	Mean (mmHg, SD)	Diastolic hypertension (DBP, ≥90 mmHg),%Ɨ	Mean (mmHg, SD)	High pulse pressure (≥55 mmHg), %Ɨ
**Gender**									
Female	687	68.4	143.3, ± 185.3	110.3, ± 15.9	9.8	73.1, ± 10.5	10.9	37.1, ± 8.8	3.3
Male	317	31.6	192.2, ± 220.8	114.0, ± 16.4	6.7	75.3, ± 11.3	12.3	38.7, ± 9.1	5.7
**Age (years)**									
30–39	405	40.3	157.5, ± 187.2	109.2, ± 13.0	2.5	72.9, ± 8.6	5.4	36.4, ± 7.0	1
40–49	263	26.2	159.5, ± 202.4	109.2, ± 15.7	5.3	72.4, ± 10.8	9.5	36.8, ± 8.7	3
50–59	156	15.6	146.5, ± 194.9	113.1, ± 16.5	10.3	74.6, ± 11.8	15.4	38.5, ± 9.0	5.1
≥60	180	17.9	171.1, ± 220.2	118.3, ± 21.5	20.6	77.3, ± 13.3	23.9	41.0, ± 11.9	11.7
BMI *(tertiles)									
<18.22	328	33	202.2, ± 224.7	106.8, ± 17.2	7	70.6, ± 11.2	7.6	36.1, ± 9.7	3.7
18.22-21.33	333	33	158.5, ± 195.8	111.4, ± 14.8	5.4	74.0, ± 10.1	10.2	37.5, ± 8.6	4.2
>21.33	343	34	117.4, ± 162.7	116.0, ± 15.0	10.5	76.7, ± 10.2	16	39.3, ± 8.3	4.4
**Education**									
No education	694	69.1	155.4, ± 199.4	111.6, ± 17.0	9.2	73.9, ± 11.3	12.5	37.8, ± 9.4	4.9
Primary	186	18.5	150.5, ± 179.7	111.0, ± 14.6	5.4	72.9, ± 10.2	10.2	38.0, ± 8.4	2.7
Secondary	124	12.4	190.0, ± 218.0	111.2, ± 12.8	2.4	74.9, ± 8.2	6.4	36.3, ± 6.8	1.6
**Religion**									
Muslim	766	76.3	196.3, ± 207.7	110.1, ± 15.9	6.1	73.0, ± 10.5	9.9	37.2, ± 9.3	3.9
Hindu	238	23.7	37.8, ± 90.2	115.7, ± 16.3	12.6	76.6, ± 11.2	16	39.2, ± 7.4	4.6
**Marital status**									
Currently married	862	85.9	160.7, ± 201.7	111.3, ± 15.6	7	73.8, ± 10.5	10.9	37.6, ± 8.7	3.7
Widowed/divorced	131	13	141.5, ± 178.4	112.5, ± 19.1	12.2	74.2, ± 12.4	14.5	38.3, ± 10.8	6.9
Never married	11	1.1	210.4, ± 167.4	110.0, ± 17.6	9.1	72.5, ± 12.4	9.1	36.5, ± 7.6	0
**Monthly income**									
≤50$ (1$ = 80tk)	537	53.5	151.0, ± 196.0	112.2, ± 17.3	10.2	74.0, ± 11.3	12.6	38.1, ± 9.5	4.7
>50$	467	46.5	167.7, ± 201.1	110.6, ± 14.6	4.7	73.6, ± 10.2	10.1	37.1, ± 8.3	3.4
**Arsenical skin lesions**									
No lesions	614	61.2	102.5, ± 154.7	113.0, ± 15.7	9	74.9, ± 10.8	13.4	72.0, ± 10.8	3.4
Melanosis	83	8.2	177.3, ± 196.4	108.1, ± 15.2	4.8	72.5, ± 9.5	7.2	35.5, ± 8.1	2.4
Keratosis	34	3.4	250.7, ± 270.0	104.1, ± 14.4	2.9	71.2, ± 10.7	2.9	32.9, ± 8.3	0
Leucomelanosis	273	27.2	268.1, ± 224.8	109.9, ± 17.0	6.2	72.0, ± 10.8	9.2	38.0, ± 11.3	6.6
* As exp. by quartile (µg/L)									
1st quartile (10–22)	296	29.5	16.5, ±4.7	112.2, ±15.0	7.8	74.9, ±10.9	10.8	37.2, ±7.4	1.7
2nd quartile (23–32)	209	20.8	28.5, ±3.5	112.1, ±17.4	10.5	74.1, ±10.6	13.9	38.0, ±9.6	4.8
3rd quartile (33–261)	253	25.2	141, ±67.7	110.4, ±16.5	5.9	73.4, ±10.9	11.1	37.0, ±8.8	4
4th quartile (≥262)	246	24.5	458.5, ±164.6	111.1, ±16.0	6.9	72.7, ±10.6	10.2	38.5, ±10.2	6.5

* SBP: Systolic blood pressure, ^*^DBP: Diastolic blood pressure; * Mean As Conc.: Mean arsenic concentration in drinking water; Ɨ: Prevalence of systolic, diastolic and pulse pressure; * As: Arsenic.

**Table 2:** Association of arsenic concentration with overall Hypertension, Systolic Hypertension, Diastolic Hypertension and Pulse pressure (n = 994).

Arsenic	Overall hypertension			Systolic hypertension ^§^			Diastolic hypertension ^ψ^			Increased pulse pressure ^φ^		
	No. of cases	Adjusted	95% CI	No. of cases	Adjusted	95% CI	No. of cases	Adjusted	95% CI	No. of cases	Adjusted	95% CI
Concentration *, (N)		OR			OR			OR			OR	
<50 (532) †	43	-	-	47	-	-	64	-	-	15	-	-
>50 (462)	23	0.93	0.49–1.78	30	1.11	0.61–2.02	50	1.24	0.76–2.01	26	3.54	1.46–8.57

* Arsenic Concentration.: Drinking water arsenic concentration in microgram/liter; **^§^** Systolic hypertension: Systolic blood pressure ≥140 mmHg; **^ψ^** Diastolic hypertension: Diastolic blood pressure ≥90 mmHg, **^φ^** Pulse pressure: categorized as <55 mmHg and ≥55 mmHg; 95% CI: 95% Confidence interval. **^†^** Reference category. Adjusted for age, sex, education, marital status, religion, monthly income and BMI. ** Crude Odds Ratios: For overall hypertension 0.60 (CI: 0.35–1.01), systolic hypertension 0.72 (CI: 0.45–1.15), diastolic hypertension 0.89 (CI: 0.60–1.31) and for increased pulse pressure 2.06 (CI: 1.07–3.93).

Overall prevalence of hypertension among the study participants was 6.6 % (male 8.2% and female 5.8%) while 12.3% of the participants reported a family history of hypertension. Systolic hypertension was identified in 7.7% (male 9.8%, female 6.7%) and diastolic hypertension in 11.3% (male 12.3%, female 10.9%) of the participants, although only 4.1% (male 5.7% and female 3.3%) had an increased pulse pressure (≥55 mmHg). Means of arsenic concentration, systolic blood pressure, diastolic blood pressure and pulse pressure by participant’s characteristics were presented in Table 1. 

In simple regression, Hindu participants were more likely to have hypertension compared to Muslims (OR 2.5, CI: 1.5–4.2). Participants with primary or high school education were 44% less likely to have hypertension than those having no formal education. There was a 5% increase in risk of having hypertension with each year increase of age and each unit increase of BMI. Table 2 shows the results of multiple regression analysis with overall hypertension by exposure to arsenic categories ≤50 μg/L and >50 μg/L, adjusted for sex, age, education, marital status, religion, monthly income, and BMI. The results shows no excess risk of overall, systolic, or diastolic hypertension for arsenic concentration >50 μg/L. Another similar adjusted multiple regression analysis by gender were performed (analysis not shown) and did not find any difference in overall and systolic hypertension but did show an association in males for diastolic hypertension (adj. OR = 3.83, 95% CI: 1.16–12.68) and in females for increased pulse pressure (adj. OR = 5.10, 95% CI: 1.59–16.38) when exposed to arsenic concentration >50 μg/L. In secondary analyses using continuous blood pressure and arsenic as continuous exposure values, results were similarly non-significant for diastolic pressure but there was a borderline positive association with arsenic and systolic pressure (coefficient 0.005, CI: −0.0 to 0.01, *P* = 0.05). However, arsenic exposure >50 μg/L was found to be strongly associated with increased pulse pressure when adjusted for other factors (OR = 3.54, 95% CI: 1.46–8.57). 

We further categorized arsenic concentrations according to quartiles and a multiple logistic regression adjusted for age, sex, education, marital status, religion, monthly income, and BMI found no effect on overall, systolic or diastolic hypertension, but did find a strong increased effect on pulse pressure (Table 3). There was an increasing trend in association with the increase of arsenic exposure levels in drinking water (*P* for trend <0.01). 

**Table 3:** Association of overall hypertension and increased pulse pressure to that of arsenic concentration by quartiles (n = 994).

As_Conc. quartile (µg/L)	N	Overall Hypertension			Pulse Pressure^ Ɨ^		
		No. of cases	Adj. OR	95% CI	No of cases	Adj. OR	95% CI
10–22 *	291	22	-	-	5	-	-
23–32	208	19	1.33	0.67–2.62	10	3.87	1.22–12.20
33–261	252	13	1.10	0.49–2.44	10	4.32	1.23–15.11
≥262	243	12	0.96	0.42–2.23	16	7.32	2.18–24.60

*P* for trend <0.01: Cuzick’s non parametric test for trend across quartiles of exposure groups; all four groups were assessed in the trend test; * Reference category. Adjusted for age, sex, education, marital status, religion, monthly income and BMI; ^Ɨ^ Pulse pressure were categorized <55 and ≥55 mmHg; ** Crude Odds Ratios: For overall hypertension, 2nd quartile 1.24 (CI: 0.65–2.36), 3rd quartile 0.67 (0.33–1.36), 4th quartile 0.64 (0.31–1.31); For increased pulse pressure, 2nd quartile 2.92 (CI: 0.98–8.68), 3rd quartile 2.39 (0.81–7.10), 4th quartile 4.04 (1.46–11.21).

Again, we categorized exposure to arsenic contaminated drinking water by ≤50 µg/L and >50 µg/L concentration and also by duration, *i.e.*, <10 and ≥10 years and performed the analysis together using all four exposure categories (≤50 µg/L and <10 years, >50 µg/L and <10 years, ≤50 µg/L and ≥10 years, and >50 µg/L and ≥10 years) adjusted for other factors. We did not find any association with overall hypertension or with systolic or diastolic hypertension or pulse pressure separately. Low dose seems to have no effect regardless of length of exposure (analysis not shown). 

Finally, we performed the analysis by ≤50 µg/L and >50 µg/L arsenic exposures in <10 years of duration and by ≤50 µg/L and >50 µg/L arsenic exposures in ≥10 years of duration to that of overall hypertension and pulse pressure separately. Results are shown in Table 4; similar to the findings in Table 2, participants exposed to arsenic concentration >50 µg/L for >10 years had an increased pulse pressure to that of participants exposed to arsenic concentration ≤50 µg/L for >10 years when adjusted for other factors. 

**Table 4:** Association of arsenic and duration of its exposure separately with overall hypertension and increased pulse pressure (n = 993).

Duration of Arsenic Exposure (years)	Arsenic Conc. (µg/L)	No. of Overall HTN ^φ^ Cases	Overall Hypertension		No. of Pulse Pressure cases	Pulse pressure ^*^	
			Adj. OR	95% CI		Adj. OR	95% CI
<10	≤50 **^†^**	12	-	-	7	-	-
	>50	10	2.05	0.61–6.87	12	2.35	0.67–8.30
≥10	≤50 **^†^**	31	-	-	8	-	-
	>50	13	0.72	0.32–1.60	14	5.25	1.41–19.51

**^φ^** HTN: overall Hypertension; ^*^ Pulse pressure was categorized as <55 and ≥55 mmHg; **^†^** Reference category. Adjusted for age, sex, education, marital status, religion, monthly income, and BMI; ^**^ Crude odds ratios in <10 years and ≥10 years group for overall hypertension were 0.79 (CI: 0.33–1.88) and 0.52 (CI: 0.27–1.01), respectively; and crude odds ratios in <10 years and ≥10 years group for increased pulse pressure were 1.69 (CI: 0.65–4.40) and 2.35 (CI: 0.97–5.68), respectively.

We also modelled a sensitivity analysis excluding participants with lesser duration of exposure (<2 and <5 years) and did not find any difference in the occurrence of overall, systolic and diastolic hypertension and pulse pressure.

## 4. Discussion

Arsenic exposure was found to be strongly associated with increased pulse pressure in our study and the association tended to be stronger in the participants with higher quartiles and longer duration of high arsenic exposure. Overall hypertension and systolic or diastolic hypertension were not associated with any level or duration of arsenic exposure in this study, except by considering systolic blood pressure and arsenic exposure as continuous variables. Our findings are similar to other studies conducted by Chen *et al.* in 2007 and by Zhang *et al.* in 2012; they observed an association between arsenic exposure and increased pulse pressure [17,42]. A recent systematic review and meta-analysis also reported an inconclusive association between arsenic and hypertension [43]. In our study, Hindu participants were more likely to be hypertensive because higher proportions of Hindus had a relatively higher weight compared to Muslims and the association between obesity and hypertension is well recognized [44]. Therefore, any association between hypertension as well as pulse pressure and arsenic exposures might be accentuated by higher BMI and confounded by participants religion. 

Increased pulse pressure in adults can occur due to atherosclerotic changes. In atherosclerosis, lacking arterial elasticity could potentially leads to arterial stiffness in the arterial walls and may subsequently increase the pulse pressure [45]. Pulse pressure is a growing concern as a risk factor for coronary artery disease and the relationship may be bidirectional [46]. One study describes pulse pressure as an independent predictive risk factor for death from cardiovascular disease, coronary heart disease, and all-cause mortality [47]. 

The important issue is how arsenic exposure could influence in increasing pulse pressure. The pathophysiology of arsenic induced increased pulse pressure is largely unknown. Possible explanations involve vascular endothelium and smooth muscles as primary targets of arsenic toxicity, which then promotes inflammatory activity [17,48]. Chronic arsenic exposure in animals can cause decreased stroke volume and cardiac output and increased vascular resistance [49]. Arsenite (trivalent arsenic or arsenic trioxide) induces oxidant accumulation and subsequently activates tyrosine phosphorylation that causes proliferation of vascular cells thereby causing cardiovascular diseases [48].

In this study, we used a large population dataset that includes arsenic concentration in individual participant’s drinking water, duration of exposure, arsenical skin lesions, and information on hypertension along with other socio-demographic factors and co-morbidity status. The study population were well described and the source of drinking water was well identified at the household level. Also, the study participants were recruited at the household level from their residing unions, so exposed and non-exposed participants were similar in their socioeconomic status, lifestyle, occupation, dietary and salt intake. 

A number of previous studies from different parts of the world including Bangladesh reported significant associations between arsenic exposure and hypertension. A recent Taiwanese study found increased risk of hypertension following chronic exposure to drinking water arsenic [50]. Tseng *et al.* reported that an individual’s capacity to complete methylation of inorganic arsenic is associated with peripheral vascular disease and hypertension [51]. In 1999, Rahman *et al.* reported a dose response relationship between drinking water arsenic and hypertension [36]. Similarly, a Chinese study also confirmed a dose response relationship with drinking water arsenic exposure and systolic blood pressure [52]. Hsueh *et al.* described a relationship between NAD(P)H oxidase, manganese superoxide dismutase (MnSOD) and endothelial nitric oxide synthase (e-NOS) polymorphisms and arsenic related hypertension [53]. Although these studies concentrated on drinking water arsenic via artesian wells, tube well or tap water, other sources of drinking water in adults such as use of bottled water and arsenic treated water might dilute the association. It is surprising that we were not able to detect an association with systolic, diastolic or overall hypertension in our study despite these previous results. Given that most of these previous studies were ecological in nature, the association may be subject to confounding [17]. 

A recent Bangladeshi study reported that arsenic exposure and cigarette smoking was synergistic for ischemic and other heart diseases [54]. However in our study, the lack of association may be due to more participation by females who were less likely to be smokers and less likely to have hypertension [55,56]. Moreover, a gender differences in biotransformation of arsenic methylation was observed previously and arsenic methylation occurred more efficiently in females [57,58]. 

Despite important public health and research implications, this study possesses some limitations. Firstly, it would have been desirable to have directly measured individual exposure data over time, because the available water samples reflected only a particular point in time and not the historical exposure. Nevertheless, in the absence of any reliable information on past exposure, it was essential to assume that arsenic concentrations from the tube wells had been relatively constant over time. The historical consistency of arsenic concentration is of particular concern with shallow ground water, which might be subject to greater fluctuation than water from a deeper well. Any fluctuation of arsenic concentrations is likely to affect the cases and controls equally. Secondly, duration of tubewell water use was self-reported recall by the participants as there was no reliable data especially on the duration of contaminated water use. Thirdly, this is a cross-sectional study and causality between drinking water arsenic and development of hypertension cannot be completely ensured. The study findings provide clues for further analytical studies. Furthermore, this study did not measure any other potential trace elements in drinking water which might have an influence on blood pressure. Further prospective epidemiologic studies in population exposed to a wide range of inorganic arsenic excluding other trace elements are needed to confirm this association, preferably in a group with individual measures of exposure over time. 

In this study, two third of the participants were female. In Bangladesh, especially in rural areas, most of the males are engaged in income generation to meet their daily requirement. As our research team visited the households during the daytime, most of the male members of the households were at work. It was therefore relevant to collect data on contraceptive pill uses by the female members because of its relationship with hypertension although use of contraceptive pills are rare in rural Bangladesh [36]. We did not find any increased prevalence of hypertension by gender, therefore contraceptive pill use is unlikely to be a confounder. 

## 5. Conclusions

Arsenic exposure from drinking water, even at lower levels (23–63 µg/L), was positively associated with high pulse pressure. A dose-response pattern was also observed between arsenic exposure as quartile and increased pulse pressure. We did not find any association between chronic arsenic exposure and overall hypertension or with systolic and diastolic hypertension separately. The study findings added further information over previous similar works. Future analytical studies would be invaluable in characterising the association and its mechanism.
